# Cannabinoid CB1 receptor agonist ACEA alleviates brain ischemia/reperfusion injury via CB1–Drp1 pathway

**DOI:** 10.1038/s41420-020-00338-3

**Published:** 2020-10-12

**Authors:** Shuai Yang, Bin Hu, Zongming Wang, Changming Zhang, Haosen Jiao, Zhigang Mao, Liguang Wei, Ji Jia, Jingling Zhao

**Affiliations:** 1 Department of Neurosurgery, the First Affiliated Hospital of Sun Yat-sen University, Guangzhou, China; 2 Department of Neurosurgery, The Second People’s Hospital of Qinzhou City, Qinzhou, China; 3 Department of Anesthesiology, General Hospital of Southern Theatre Command of PLA, Guangzhou, China; 4 Department of Burns, the First Affiliated Hospital of Sun Yat-sen University, Guangzhou, China

**Keywords:** Neurological disorders, Cell death

## Abstract

Activation of the cannabinoid CB1 receptor induces neuroprotection against brain ischemia/reperfusion injury (IRI); however, the mechanism is still unknown. In this study, we used oxygen-glucose deprivation/reoxygenation (OGD/R)-induced injury in neuronal cells and middle cerebral artery occlusion (MCAO)-induced brain IRI in rats to mimic ischemic brain injury, and hypothesized that the CB1 receptor agonist arachidonyl-2-chloroethylamide (ACEA) would protect ischemic neurons by inhibiting mitochondrial fission via dynamin-related protein 1 (Drp1). We found that OGD/R injury reduced cell viability and mitochondrial function, increased lactate dehydrogenase (LDH) release, and increased cell apoptosis, and mitochondrial fission. Notably, ACEA significantly abolished the OGD/R-induced neuronal injuries described above. Similarly, ACEA significantly reversed MCAO-induced increases in brain infarct volume, neuronal apoptosis and mitochondrial fission, leading to the recovery of neurological functions. The neuroprotective effects of ACEA were obviously blocked by coadministration of the CB1 receptor antagonist AM251 or by the upregulation of Drp1 expression, indicating that ACEA alleviates brain IRI via the CB1–Drp1 pathway. Our findings suggest that the CB1 receptor links aberrant mitochondrial fission to brain IRI, providing a new therapeutic target for brain IRI treatment.

## Introduction

Stroke is the second leading cause of death worldwide, but in China, stroke is the leading one. Unfortunately, medications for preventing and treating stroke are very limited. Currently, only recombinant tissue plasminogen activator (rTPA) have been proven to be effective. Because of the narrow therapeutic window (within 4.5 h after the onset of stroke), only 3–8.5% of new stroke patients can receive rTPA treatment^1^. Therefore, exploring novel medications and therapies for stroke is highly urgent and important.

Cannabinoid receptors are expressed in the brain tissue^2^. Numerous studies have shown that cannabinoid receptor activation induces neuroprotection against ischemic brain injury^3–5^; however, the exact mechanism is still unknown^6–8^. At present, two main cannabinoid receptors have been discovered, CB1 and CB2. In the central nervous system (CNS), the CB1 receptor is mainly expressed in neurons and astrocytes, and the CB2 receptor is located in microglial cells and astrocytes^9,10^. Previous observations from us and other researchers indicated that the CB1 agonist arachidonyl-2-chloroethylamide (ACEA) protects ischemic brain tissue by ameliorating neuronal mitochondrial functions^3,11,12^, but how the process occurs is unknown. Mitochondrial fission is regarded as an early event in mitochondrial apoptosis^13,14^; therefore, the inhibition of neuronal mitochondrial fission is considered to be a key strategy to preserve mitochondrial function and maintain neurological ability^15,16^.

In neurons, the CB1 receptor is expressed in the plasma membrane and mitochondria^17,18^. Dynamin-related protein 1 (Drp1) is a key protein that regulates mitochondrial fission after brain ischemia^19^. Many studies have shown that downregulating Drp1 can induce neuroprotection after stroke^20,21^. To explore the potential molecular mechanisms of CB1 receptor activation, in the present study, we used oxygen-glucose deprivation/reoxygenation (OGD/R) and middle cerebral artery occlusion (MCAO) in vitro and in vivo, respectively, as brain IRI models and examined whether the CB1 agonist ACEA protects ischemic brain tissue via the CB1–Drp1 pathway by inhibiting mitochondrial fission.

## Results

### The CB1 receptor agonist ACEA dose-dependently reduced OGD/R-induced cell injury

**Fig. 1:**
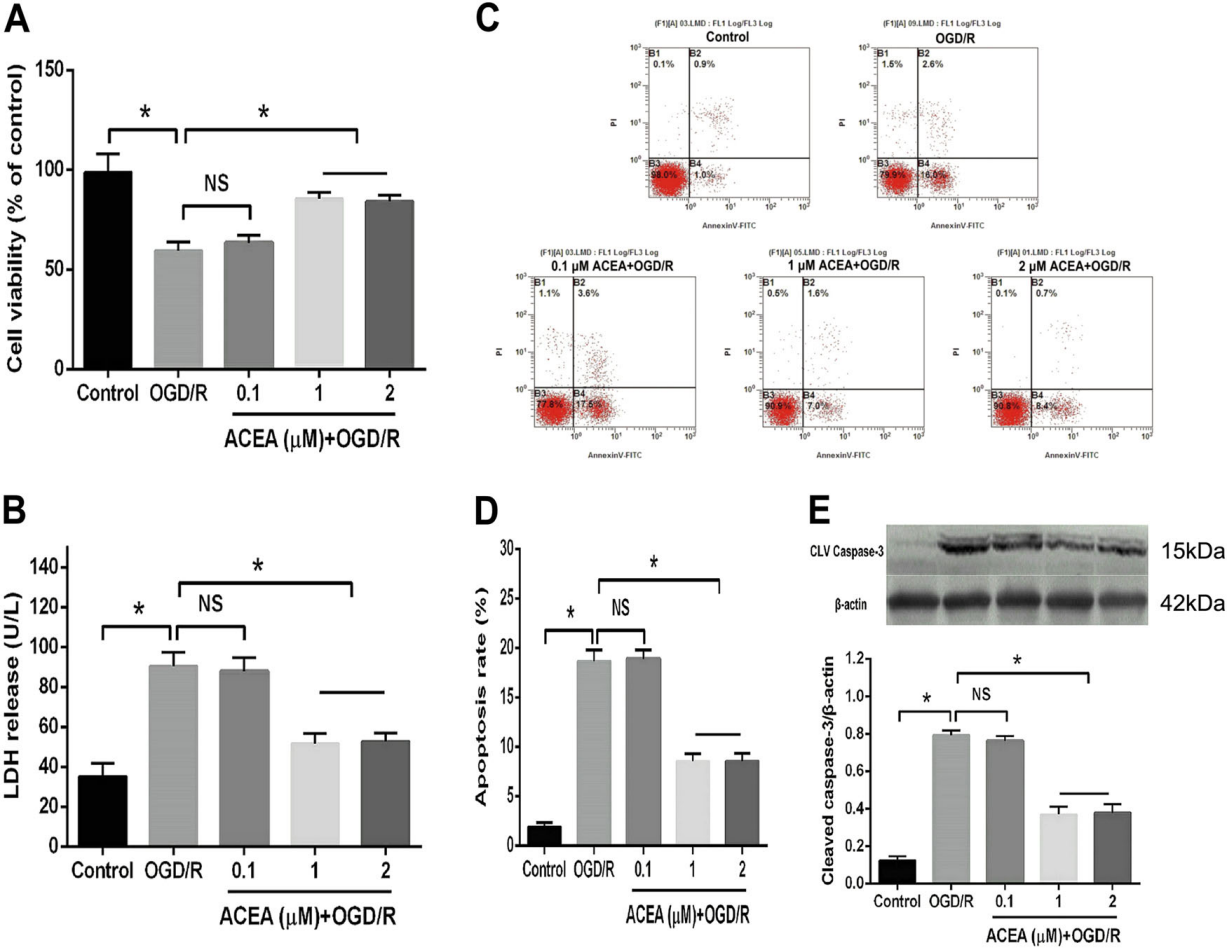
The CB1 cannabinoid receptor agonist ACEA alleviated OGD/R-induced injury in HT22 cells. **A** ACEA restored cell viability in HT22 cells exposed to OGD/R. **B** ACEA decreased lactate dehydrogenase (LDH) release from HT22 cells. **C** The ACEA-induced effects on cell apoptosis in OGD/R-induced HT22 cells were measured by flow cytometry. **D** ACEA inhibited cell apoptosis in OGD/R-induced HT22 cells. **E** ACEA reduced cleaved caspase-3 expression in OGD/R-induced HT22 cells. *p < 0.05; NS no significance.

To observe the protective effects of the CB1 cannabinoid receptor agonist ACEA on OGD/R-induced HT22 cells, the cells were divided into five groups: the control group, the 4 h of OGD plus 24 h of reoxygenation (OGD/R) group and three ACEA concentrations (0.1, 1, and 2 μM) plus OGD/R stimulation groups. Compared with that of the control group, OGD/R obviously decreased cell viability (Fig. 1A) and increased LDH release (Fig. 1B), the apoptosis rate (Fig. 1C, D) and the expression of cleaved caspase-3 (Fig. 1E), which is an apoptosis-associated protein. However, administration of 1 or 2 μM ACEA significantly ameliorated the cell viability and decreased LDH release, cell apoptosis and cleaved caspase-3 expression. ACEA at 0.1 μM did not exhibit obvious protective effects in the OGD/R-induced cells.

### AM251 but not hemopressin reversed the ACEA-induced effects on mitochondrial fission in OGD/R-induced neuronal cells

**Fig. 2:**
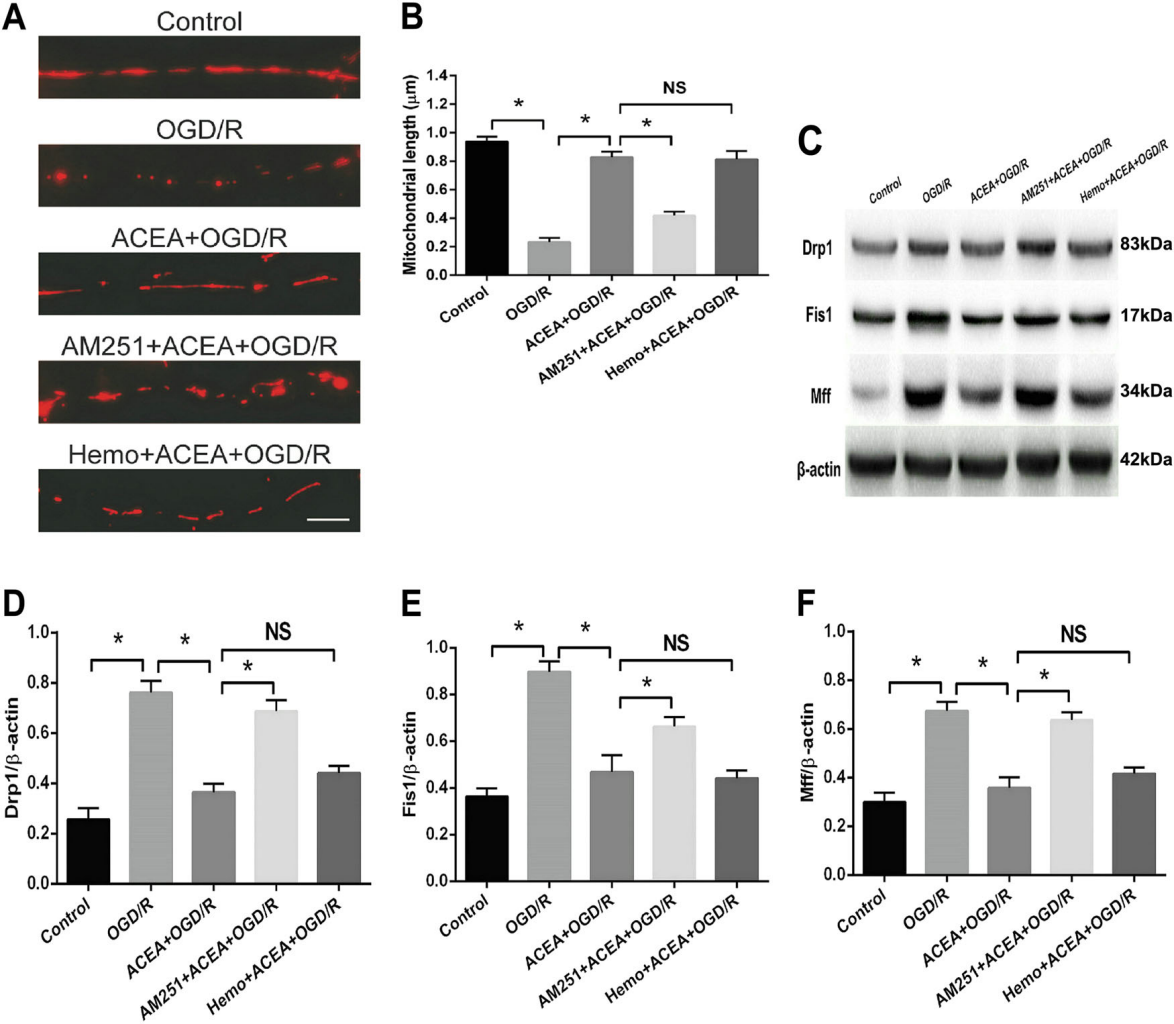
The CB1 receptor antagonist AM251 reversed the ACEA-induced effects on mitochondrial fission and the expression of fission-associated proteins. **A** Representative images of neuronal dendrites expressing Mito-DsRed, a mitochondrial marker. **B** Statistical results of the images in (**A**)**. C** Western blot results showing the ACEA-induced effects on Drp1, Fis1, and Mff expressions. **D** AM251 reversed the ACEA-induced downregulation of Drp1 expression in OGD/R-induced HT22 cells. **E** AM251 reversed the ACEA-induced downregulation of Fis1 expression. **F** AM251 reversed the ACEA-induced downregulation of Mff expression. Bar = 1 µm; *p < 0.05; NS no significance.

AM251 and hemopressin (Hemo) are two CB1 receptor antagonists, and the difference between the two antagonists is that AM251 can penetrate the cell membrane and reverse CB1 agonist-induced effects at both the cell membrane and mitochondria; in contrast, Hemo cannot penetrate the cell membrane; therefore, Hemo can only reverse CB1 agonist-induced effects on the cell membrane but not mitochondria^22^. To evaluate the effect of the CB1 agonist ACEA on mitochondrial fission, mitochondrial lengths were measured. As shown in Fig. 2A, B, OGD/R significantly shortened the lengths of mitochondria, which were restored by ACEA treatment. However, AM251 but not Hemo obviously reversed the effect of ACEA, shorting the lengths of mitochondria in the presence of ACEA. In addition, we examined the expression of mitochondrial fission-associated proteins, including Drp1, Fis1, and Mff. Our results showed that OGD/R increased the expression of these three proteins, whereas ACEA treatment decreased their expression. AM251 but not Hemo, reversed the effect of ACEA, leading to increases in the expression of these three proteins (Fig. 2C–F). These results indicated that activation of the mitochondrial CB1 receptor inhibited OGD/R-induced mitochondrial fission.

### Drp1 upregulation abolished ACEA-induced cytoprotection in OGD/R-induced neuronal cells

**Fig. 3:**
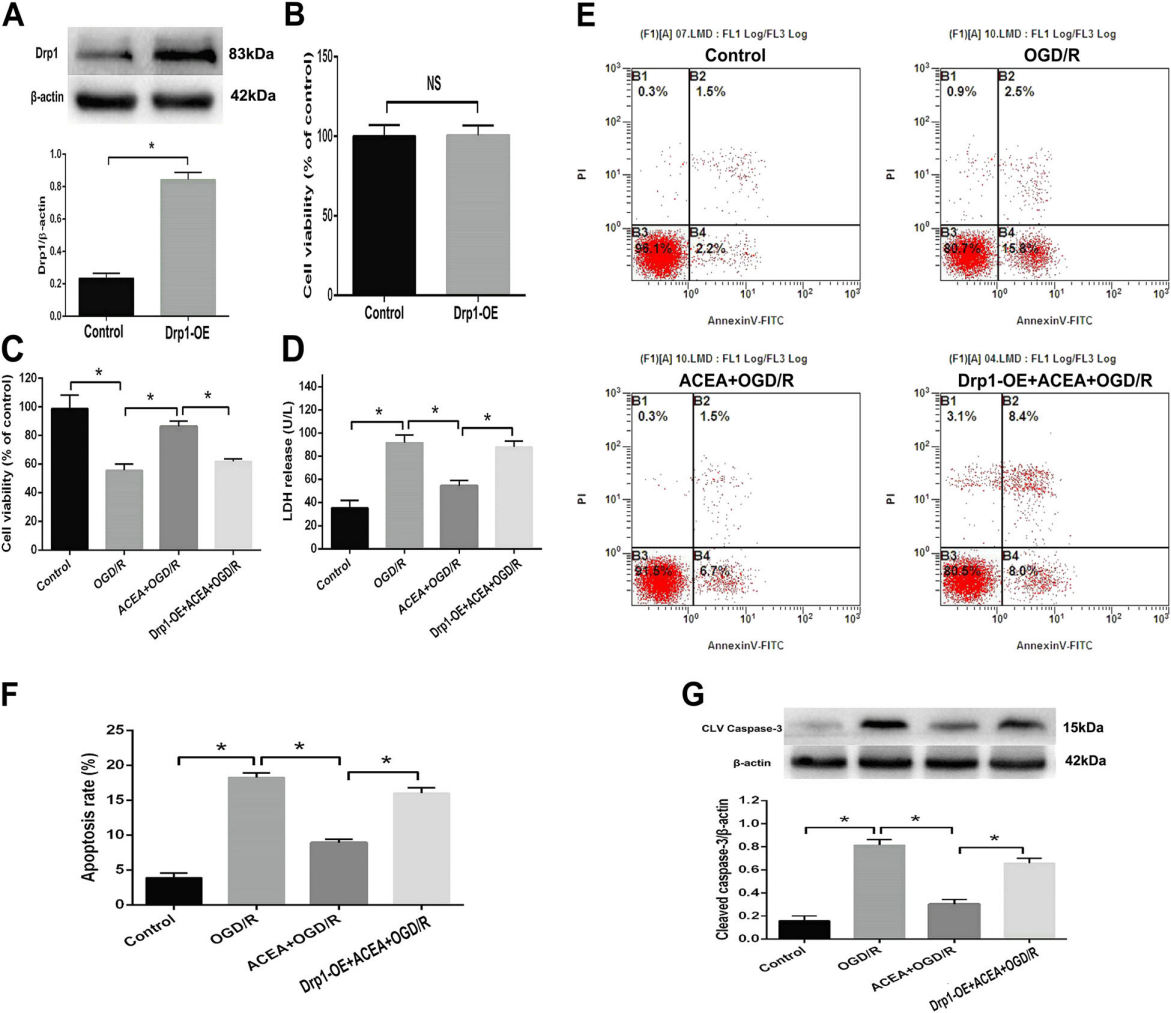
Drp1 overexpression abolished ACEA-induced cytoprotection in HT22 cells exposed to OGD/R. **A** The Drp1 wt vector induced Drp1 protein overexpression (Drp1-OE) in HT22 cells. **B** Drp-OE did not induce obvious cytotoxicity in HT22 cells. **C** Drp1-OE reversed the cannabinoid CB1 agonist ACEA-induced restoration of cell viability. **D** Drp1-OE reversed the ACEA-induced inhibition of LDH release. **E** The Drp1-OE-induced effects on cell apoptosis were measured by flow cytometry. **F** Drp1-OE reversed the ACEA-induced inhibition of cell apoptosis. **G** Drp1-OE reversed the ACEA-induced effect on cleaved caspase-3 expression. *p < 0.05; NS no significance.

To investigate the role of Drp1 in ACEA-induced protection against OGD/R injury in HT22 cells, a Drp1wt vector clone was used to induce overexpression of the Drp1 protein. The transfection efficiency was confirmed by western blot analysis; the results showed that the expression of Drp1 observably increased (Fig. 3A), and the cell viability was not changed after transfection (Fig. 3B), indicating that overexpression of Drp1 in HT22 cells did not induce cytotoxicity. In addition, the ACEA-induced increase in cell viability and reductions in LDH release, cell apoptosis, and cleaved caspase-3 expression under OGD/R conditions were abolished by Drp1 overexpression, suggesting that ACEA-induced protection against OGD/R in HT22 cells by inhibiting the expression of Drp1.

### Drp1 upregulation abolished the ACEA-induced mitochondrial fission and dysfunctions

**Fig. 4:**
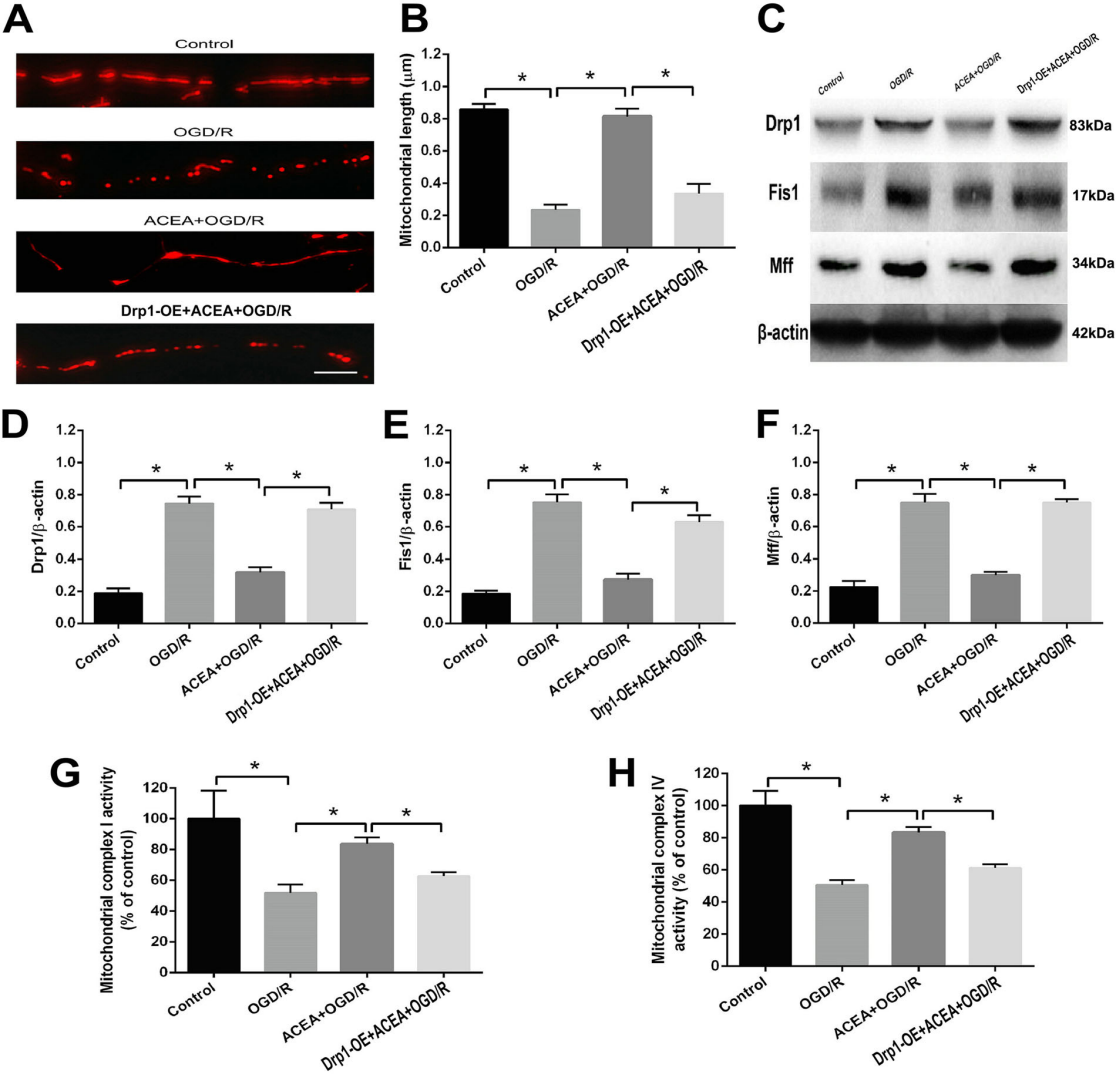
Drp1 overexpression abolished the ACEA-induced effects on mitochondrial fission, fission-associated protein expression, and mitochondrial function in HT22 cells exposed to OGD/R. **A** Representative images of neuronal dendrites expressing Mito-DsRed, a mitochondrial marker. **B** Statistical results of the images in (**A**). **C** Western blot results showing Drp1, Fis1, and Mff expressions. **D** Drp1 overexpression (Drp1-OE) abolished the cannabinoid CB1 agonist ACEA-induced effect on Drp1 expression. **E** Drp1-OE abolished the ACEA-induced effect on Fis1 expression. **F** Drp1-OE abolished the ACEA-induced effect on Mff expression. **G**, **H**. Drp1-OE reversed the ACEA-induced effects on the activities of mitochondrial complexes I and IV in HT22 cells. Bar = 1 µm; *p < 0.05; NS no significance.

To further determine the role of Drp1 in ACEA-induced mitochondrial protection, mitochondrial lengths, the levels of mitochondrial fission-associated proteins and the activities of mitochondrial complex I and complex IV were analyzed. As shown in Fig. 4A, B, under OGD/R conditions, ACEA increased mitochondrial lengths, which were reversed after overexpression of Drp1. Moreover, the ACEA-induced reductions in Drp1, Fis1, and Mff expression under OGD/R conditions was significantly increased after Drp1 overexpression (Fig. 4C–F). In addition, the activities of mitochondrial complexes I and IV, which were reduced under OGD/R conditions, were obviously increased after ACEA treatment. However, Drp1 overexpression abolished the effects of ACEA and decreased the activities of mitochondrial complexes I and IV (Fig. 4G, H).

### AM251 but not hemopressin reversed ACEA-induced neuroprotection against brain IRI in rats

**Fig. 5:**
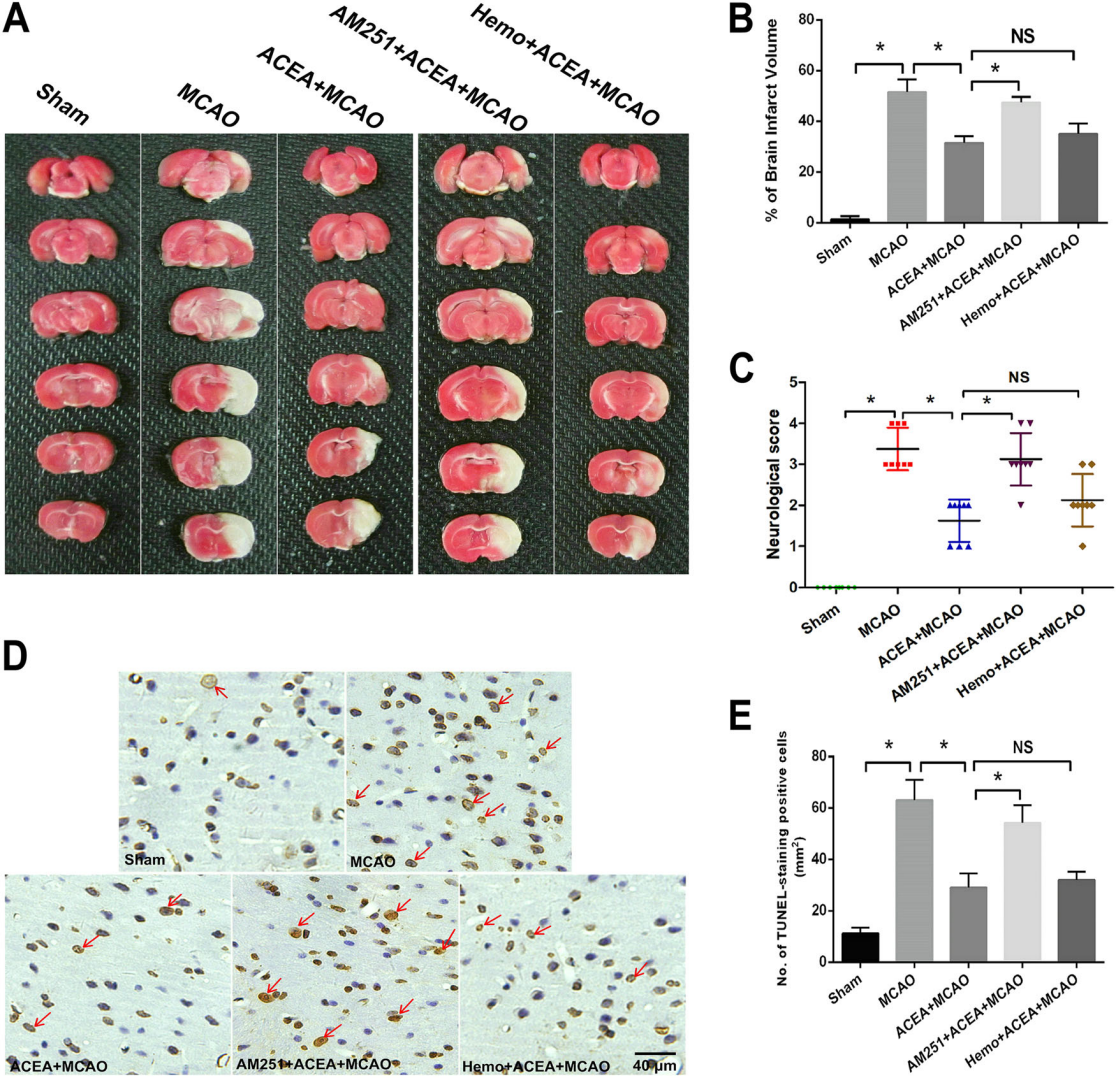
The cannabinoid CB1 receptor agonist ACEA reduced ischemic brain injury in rats. **A**TTC staining of rat brain slices. **B** The CB1 receptor antagonist AM251 reversed the ACEA-induced effect on brain infarct volume in rats. **C** AM251 reversed the ACEA-induced amelioration of neurological function in rats. **D** TUNEL staining of brain tissue. **E** AM251 reversed the ACEA-induced inhibition of cell apoptosis. Bar = 40 µm; *p < 0.05; NS no significance.

To evaluate the protective effect of the CB1 receptor agonist ACEA in vivo, a middle cerebral artery occlusion (MCAO) model was established in rats to mimic brain IRI. As shown in Fig. 5A, B, the increased brain infarct volume induced by MCAO was decreased after ACEA treatment. However, the CB1 antagonist AM251, but not Hemo, reversed the ACEA-induced protective effects and obviously increased the infarct volume. Moreover, the MCAO-induced increase in neurological score (a higher score indicated impaired neurological function) was reduced by ACEA but was increased after treatment with AM251 but not Hemo (Fig. 5C). In addition, the neuronal apoptosis level, which was increased in the MCAO group, was decreased after ACEA treatment. The application of AM251 but not the Hemo, significantly reversed the effect of ACEA (Fig. 5D, E). These results indicated that activation of the mitochondrial CB1 receptor exerted neuroprotective effects against ischemic brain injury in vivo.

### AM251 but not hemopressin reversed the ACEA-induced effects on mitochondrial CB1 expression and fission

**Fig. 6:**
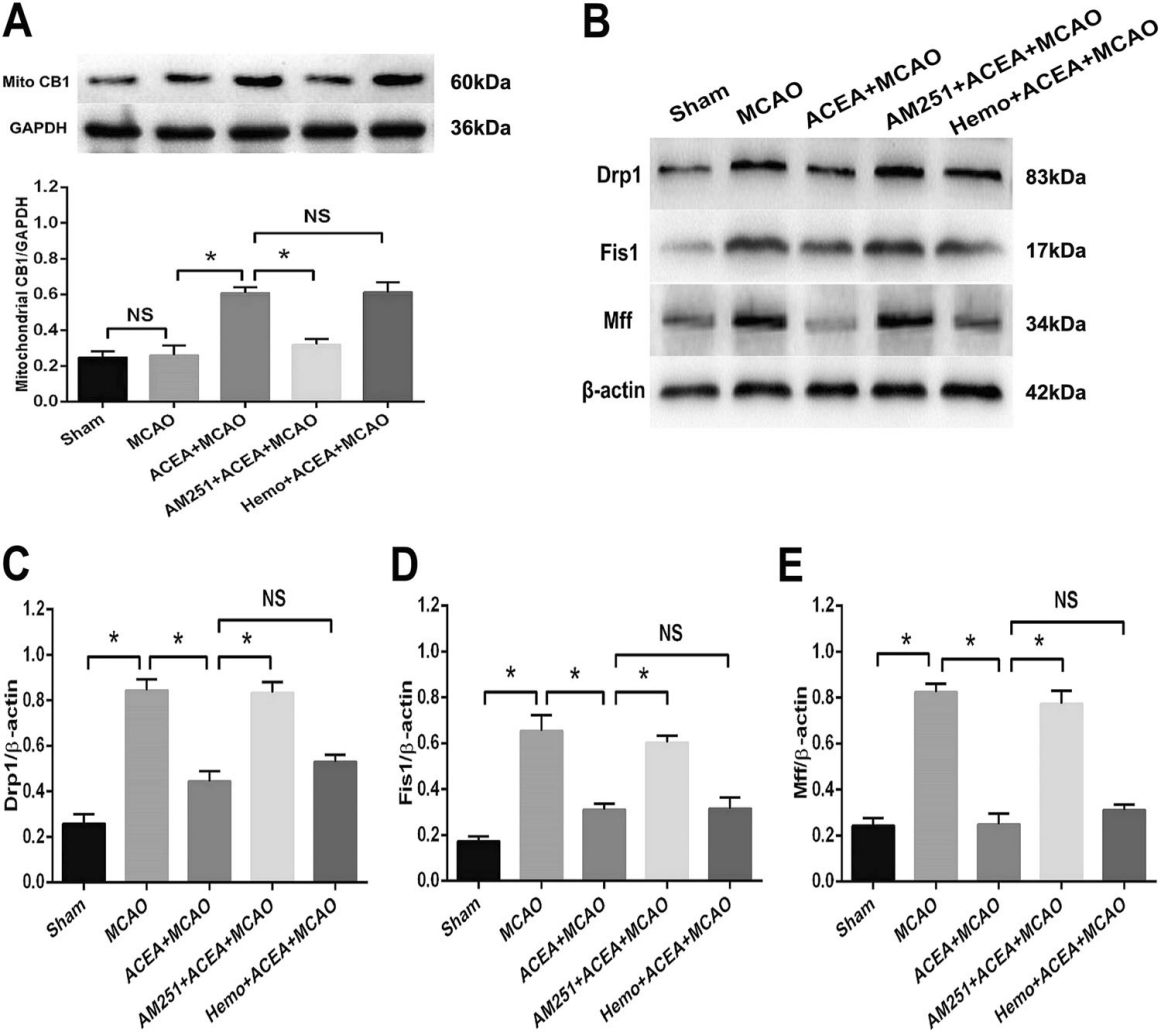
The CB1 cannabinoid receptor agonist ACEA reduced the expression of mitochondrial fission-associated proteins in rats after ischemic brain injury. **A** The CB1 antagonist AM251 reversed the ACEA-induced upregulation of mitochondrial CB1 expression. **B** Western blot results of Drp1, Fis1 and Mff expression in rats. **C** AM251 reversed the ACEA-induced inhibition of Drp1 expression in rats after brain ischemia. **D** AM251 reversed the ACEA-induced inhibition of Fis1 expression. **E** AM251 reversed the ACEA-induced inhibition of Mff expression. *p < 0.05; NS no significance.

We next evaluated the role of the CB1 cannabinoid receptor in regulating mitochondrial fission in vivo. As shown in Fig. 6A, ACEA increased the protein expression of mitochondrial CB1, and AM251 but not hemopressin, abolished the ACEA-induced upregulation of mitochondrial CB1 expression. Moreover, MCAO increased the expression of mitochondrial fission-associated proteins, including Drp1, Fis1, and Mff, in ischemic brain tissue. The ACEA treatment significantly reduced the expression of the three proteins, and these effects were abolished by the CB1 antagonist AM251 but not Hemo (Fig. 6B–E). These findings indicated that mitochondrial CB1 receptor activation could inhibit mitochondrial fission in ischemic brain tissue.

## Discussion

In this study, we found that the CB1 cannabinoid receptor agonist ACEA inhibited OGD/R-induced mitochondrial fission by decreasing the expression of Drp1, leading to increased cell viability and mitochondrial functions and reduced LDH release and cell apoptosis. An in vivo study further demonstrated that ACEA inhibited the MCAO-induced increases in brain infarct volume, neuronal apoptosis, and mitochondrial fission, exerting neuroprotective effects against brain IRI.

Neurons are sensitive to ischemia, and neuroregeneration is extremely difficult in injured brain tissue. Thus, it is important to find effective therapeutic strategies for the treatment of ischemic brain injury. Among the multiple pathogenesis associated with brain IRI, such as excess inflammatory responses, oxidative stress injury, cellular oxidation, neuronal apoptosis and autophagy^23^, mitochondrial fission and dysfunction are believed to be key pathological characteristics. Mitochondria are the energy factory of cells, and mitochondrial impairment in the CNS induces neuronal injury and death, ultimately leading to neurological dysfunction. Therefore, maintaining neuronal mitochondrial function could be a promising therapeutic strategy for treating stroke^24^. Cannabinoid receptors are expressed in brain tissue, and activation of these receptors give rise to neuroprotection against brain IRI. In our previous studies, we found that activation of the CB1 cannabinoid receptor by ACEA decreased brain IRI by restoring mitochondrial functions, and inhibition of mitochondrial permeability transition pore (MPTP) opening may be involved in the neuroprotective effects of ACEA^3,25^. Since the opening of the MPTP and subsequent mitochondrial swelling and enlargement lead to mitochondrial fission, we hypothesized that ACEA protects neuronal function in brain IRI by regulating mitochondrial fission.

As a physiological response that occurs in numerous biological processes, mitochondrial fission also occurs after brain IRI. Normal mitochondrial fission provides additional daughter mitochondria to meet the energy requirements of the cell^26,27^. However, excessive mitochondrial fission produces a large amount of mitochondrial fragments, which are harmful to the homeostasis and functions of cells. Excessive mitochondrial fission has been reported to be involved in brain IRI, serving as a primary contributor to cerebral IRI-induced neuronal death^28^. In this study, by observing mitochondrial length and measuring the expression of mitochondrial fission-associated proteins^29^, we found that activation of the cannabinoid CB1 receptor using ACEA reduced OGD/R- or brain ischemia-induced mitochondrial fission and dysfunction. The CB1 receptor is located both in the neuronal membrane and in mitochondria. To investigate which receptor was involved in protecting neuronal function under OGD/R conditions, AM251, a lipophilic cell-permeant CB1 antagonist that can penetrate the plasma membrane, and hemopressin, a cell-impermeant CB1 receptor antagonist that is unable to penetrate the plasma membrane^30,31^, were used. We found that AM251 but not hemopressin reversed ACEA-induced neuroprotection, indicating that the mitochondrial CB1 receptors but not the cell membrane CB1 receptors mediated mitochondrial fission, thus exerting neuroprotective effects against brain IRI. As highly dynamic organelles, mitochondrial fusion and fission are tightly regulated by specialized proteins. Among these mitochondria-shaping proteins, Drp1 is regarded as the main profission protein^19^. The activity of Drp1 is tightly regulated to ensure strict control over mitochondrial shape according to cell needs^32^. Drp1-mediated mitochondrial fission has been reported to be involved in the pathogenesis of brain IRI. Studies have shown that the genetic disinhibition of Drp1 via knockout of the mitochondrial protein kinase A (PKA) scaffold AKAP1 exacerbates brain IRI in mice^33^. Administration of Drp1 inhibitors, such as mdivi-1, inhibit excessive mitochondrial fission, thus alleviating the neurological injury induced by cerebral I/R^15^. However, direct inhibition of Drp1 may cause obvious adverse effects^34^, as postnatal genetic ablation of Drp1 in the forebrain leads to synaptic dysfunction and cognitive impairment. Recently, CB1 receptor activation was reported to induce cytoprotection against oxidative injury by modulating Drp1 phosphorylation levels in mice^35^. In this study, we found that Drp1-mediated mitochondrial fission was involved in the neuroprotective effects of CB1 cannabinoid receptor activation against brain IRI. By inhibiting Drp1, ACEA reduced mitochondrial fission, increased cell viability and neurological function, and exerted a neuroprotective effect against brain IRI.

Overall, our study demonstrated that the CB1 cannabinoid receptor agonist ACEA alleviates OGD/R- or brain ischemia-induced neuronal injury by regulating CB1–Drp1-mediated mitochondrial fission.

## Materials and methods

### Cells, rats, and reagents

HT22 cells were obtained from the Fourth Military Medical University. Sprague-Dawley (SD) rats were purchased from the Laboratory Animal Center of Sun Yat-sen University. The CB1 cannabinoid receptor agonist ACEA, CB1 receptor antagonist AM251 (can penetrate the cell membrane), cell membrane CB1 receptor antagonist hemopressin (Hemo), 3-(4,5-dimethyl-2-thiazolyl)-2,5-diphenyl-2-H-tetrazolium bromide (MTT), dimethyl sulfoxide (DMSO), DMEM cell culture medium and fetal bovine serum (FBS) were purchased from Sigma-Aldrich (St. Louis, MO, USA).

### Cell culture and OGD/R treatment

HT22 cells were cultured in DMEM containing 10% FBS (v:v), 100 U/ml penicillin and 100 μg/ml streptomycin. The incubator contained 95% O_2_ and 5% CO_2_ at 37 °C. The medium was changed every 2–3 d, and the cells were passaged twice per week at a split ratio of 1:4.

For the OGD and reoxygenation treatment, the cells were seeded into a cell culture plate, and after 24 h of incubation in normal medium, the cell culture medium was changed to medium containing no glucose or FBS. In addition, the air in the container contained 95% N_2_ and 5% CO_2_ by volume. After 4 h of incubation in the medium at 37 °C, the medium was changed to normal medium containing glucose and 10% FBS. After 24 h of reoxygenation, cell injury, mitochondrial length and protein expression were assessed.

### Drp1 protein overexpression

The pCMV6-MycDDK-Drp1wt vector was purchased from OriGene Technologies, Inc. (Rockville, MD, USA). HT22 cells were transfected with the pCMV6-MycDDK-Drp1wt vector for 2 d as shown in a previous study^29^. Drp1 expression was evaluated by using western blot analysis.

### Experimental protocols

To determine a suitable ACEA concentration in vitro, HT22 neuronal cells were divided into five groups, including the Control group, the 4 h of OGD plus 24 h of reoxygenation group (OGD/R), and three ACEA treatment groups (cells were treated with 0.1, 1 or 2 μM ACEA for 4 h and then exposed to OGD/R). After the treatments, cell injury and apoptosis levels were evaluated. Then, to investigate the role of the CB1 receptor in ACEA-induced neuroprotection, the cells were divided into five groups: Control, OGD/R (4 h of OGD plus 24 h of reoxygenation), 1 μM ACEA + OGD/R, 10 μM cell-permeant CB1 receptor antagonist AM251 + ACEA + OGD/R, and 10 μM cell-impermeant CB1 receptor antagonist hemopressin + ACEA + OGD/R. After the treatments, mitochondrial length and mitochondrial fission-associated proteins were evaluated. To assess the role of Drp1 in ACEA-induced neuroprotection, the cells were divided into four groups: Control, OGD/R (4 h of OGD plus 24 h of reoxygenation), 1 μM ACEA + OGD/R, and Drp1-OE + ACEA + OGD/R (cells were transfected with the pCMV6-MycDDK-Drp1wt vector for 2 d and treated with 1 μM ACEA and OGD/R). After the treatments, cell injury levels, apoptosis, mitochondrial fission levels and mitochondrial functions were evaluated.

To further observe the role of CB1 in ACEA-induced neuroprotection and mitochondrial fission-associated protein expression in vivo, SD rats were assigned into five groups: sham (underwent the operation without brain ischemia), MCAO (regional brain ischemia for 1.5 h and reperfusion for 24 h), ACEA + MCAO (the rats were administered 1.5 mg/kg ACEA intraperitoneally, and 30 min later, the rats underwent 1.5 h of MCAO and 24 h of reperfusion), AM251 + ACEA + MCAO (the rats were simultaneously injected with 1 mg/kg AM251 and 1.5 mg/kg ACEA intraperitoneally, and 30 min later, the rats underwent 1.5 h of MCAO and 24 h of reperfusion), and Hemo+ACEA + MCAO (the rats were simultaneously injected with 1 mg/kg hemopressin and 1.5 mg/kg ACEA intraperitoneally, and 30 min later, the rats underwent 1.5 h of MCAO and 24 h of reperfusion). After the treatments, brain volume, neurological function, neuronal apoptosis and the expression of mitochondrial fission-associated proteins were evaluated. The sample size in each group is six.

### Cell viability and LDH release assays

HT22 cells were seeded into a 96-well cell culture plate, and after the treatments, 20 μl of MTT solution was added into each well. After incubation at 37 °C for 4 h, the cell culture medium of each well was removed. Then, 150 μl of DMSO was added into each well. After the formazan in each well was dissolved completely, the absorbance of each well was measured by using a spectrophotometer at a wavelength of 490 nm.

The cells were plated into a 24-well cell culture plate. After the treatments, the supernatant of each well was collected, and the LDH activity in the supernatant was determined by using an LDH reagent kit as previously described^3^.

### Cell apoptosis assay

Flow cytometry (BD, USA) was used to measure the cell apoptosis rate. The cells were seeded into a 6-well plate at a density of 2 × 10^5^ cells/well, and after the treatments, the cells were harvested by centrifugation. After being washed with ice-cold phosphate-buffered saline (PBS), the cells were resuspended in binding buffer. Then, fluorescein 5-isothiocyanate [2-(3,6-dihydroxy-9H-xanthen-9-yl)-5-isothiocyanatobenzoic acid] FITC-conjugated anti-annexin-V staining antibody and propidium iodide solution were added to the binding buffer. After the cells and the buffer were mixed completely, the cells were incubated at room temperature for 15 min in the dark, and the cell apoptosis rate was assessed.

### Western blot analysis

The in vitro and in vivo protein levels were evaluated by using the Bradford method. Western blot procedures were performed as previously described^3^. We used the following primary antibodies: anti-cleaved caspase-3, anti-Drp1, anti-Fis1, anti-Mff, anti-CB1, and anti-β-actin (1:200 in dilution). Suitable horseradish peroxidase-conjugated goat anti-rabbit secondary antibodies (CWBIO, Beijing, China) were used. Image analysis was performed with the assistance of computerized analysis software (Bio-Rad Laboratories, Hercules, CA).

### Mitochondrial isolation complex activity assay

In vivo, rats were anesthetized and then decapitated, and the brains were rapidly removed. The penumbra region was removed and used for mitochondrial isolation. In vitro, after the treatments, the cells were exposed to trypsin for 1–2 min and then centrifuged and harvested for mitochondrial isolation. Mitochondria were isolated according to the manufacturer’s specifications (Qproteome mitochondria isolation kit, Qiagen, Hilden, Germany). The activities of mitochondrial complexes I and IV were determined spectrophotometrically at 30 °C by the methods described in a previous study^11^.

### Ischemic brain injury model

Focal cerebral ischemia/reperfusion injury was performed as described previously^28^. The treatment of the animals conformed to the Committee on Publication Ethics guidelines. The protocol was approved by the Committee on the Ethics of Animal Experiments of Sun Yat-sen University. Briefly, the rats were anesthetized with 1.5% isoflurane mixed with oxygen, and ischemia-reperfusion was induced by 30 min of right middle cerebral artery occlusion with a 7.0 siliconized filament (Doccol, USA), followed by a 24 h of reperfusion. During the operation, the body temperature of the rats was maintained at 37.0 °C ± 1.0 by using a thermostatic pad. Laser Doppler flowmetry (Perimed Instruments, USA) was used for each rat to ensure that regional cerebral blood flow was decreased by 80–90% and recovered to 80–95% of baseline after removal of the filament, indicating adequate cerebral ischemia and reperfusion. Six rats in each group, and the animals were randomly grouped.

### Neuronal culture and transfection

HT22 cells were plated on coverslips at a density of 5 × 10^4^ cells/coverslip in DMEM containing 10% FBS. The coverslips had been precoated overnight at 37 °C with poly-L-lysine. After the treatments, the cell culture medium was replaced with serum-free DMEM. The serum-free medium contained 0.5 mg/ml Lipofectamine 2000 and the Mito-DsRed plasmid (pCKII-Mito-DsRed). After being transfected for 24 h, the cells were washed 3 times with PBS in the dark at room temperature, and then photos were taken by using a confocal microscope (Olympus, Japan).

### TUNEL staining

Twenty-four hours after reperfusion, neuronal apoptosis in the ischemic penumbra was measured in situ by using terminal deoxynucleotide transferase-mediated dUTP nick-end labeling (TUNEL) staining as previously described^11^. Briefly, regions of interest were analyzed by light microscopy, and then the total number of positively stained cells in these regions was counted and expressed as cells per mm^2^.

### Neurobehavioral evaluation and infarct measurement

Twenty-four hours after reperfusion, a 5-point scoring system modified from Longa was used by a blinded observer to evaluate neurological deficiency. The rating scale was as follows: 0 = no deficit; 1 = failure to extend the left forepaw; 2 = decreased grip strength of the left forepaw; 3 = circling to the left by pulling the tail; and 4 = spontaneous circling. The rats were deeply anesthetized by using 1.5% pentobarbital sodium (30 mg/kg) via intraperitoneal injection, the brains were removed, and the infarct volume was measured as previously described. The brain sections were stained with 2% TTC solution for 10 min at 37 °C, followed by treatment with 4% paraformaldehyde. The stained sections were photographed with a digital camera. Unstained areas were defined as infarcts and were measured using image analysis software (Adobe Photoshop CS3 12.0 for Windows) by an investigator who was blinded to the experimental groupings. Infarct volume was quantified as follows: relative infarct volume = (contralateral area-ipsilateral noninfarcted area)/contralateral area.

### Statistical analysis

SPSS 13.0 for Windows (SPSS Inc., Chicago, IL) was used to conduct statistical analyses in this investigation. Except for the neurological scores, all values in this study are expressed as the mean ± standard deviation (S.D.). Differences between groups were compared by using one-way ANOVA followed by Tukey’s multiple comparisons test. The neurological scores are expressed as the median (interquartile range) and were analyzed by using the Kruskal–Wallis test followed by the Mann–Whitney *U* test with Bonferroni correction. The investigators are double-blinded. A value of *P* < 0.05 indicated significance.
